# Malignant Hypercalcemia: A Rare Etiology of Posterior Reversible Encephalopathy Syndrome

**DOI:** 10.7759/cureus.41229

**Published:** 2023-06-30

**Authors:** Victor A Odoma, Iman Zahedi, Hassan Haq, Stefany C Lopez Pantoja, Ezrah C Onyejide, Farzana Rahman

**Affiliations:** 1 Cardiology/Oncology, Indiana University (IU) Health, Bloomington, USA; 2 Internal Medicine, Arrowhead Regional Medical Center, Los Angeles, USA; 3 Medicine, Jinnah Sindh Medical University, Karachi, PAK; 4 Internal Medicine, Pontifical Catholic University of Ecuador, Chagrin Falls, USA; 5 Family Medicine, Metropolitan University College of Medicine, St. John’s, ATG; 6 Internal Medicine, Jalalabad Ragib Rabeya Medical College and Hospital, Sylhet, BGD

**Keywords:** pres syndrome, malignant hypercalcemia, severe hypercalcemia, advanced breast carcinoma, posterior reversible encephalopathy syndrome (pres)

## Abstract

Posterior reversible encephalopathy syndrome (PRES) is a rare and severe neurotoxic encephalopathic state characterized by variable neurologic manifestations ranging from headache and confusion to seizures, coma, and reversible subcortical vasogenic edema on imaging. PRES is commonly induced by chronic renal failure, hypertension, chemotherapeutic drugs, and eclampsia. PRES induced by hypercalcemia is uncommon and not widely underlined in the literature. We underline a case of a 61-year-old female diagnosed with advanced breast carcinoma presented with altered sensorium and generalized limb weakness. She was found to have malignant hypercalcemia, and brain imaging demonstrated subcortical vasogenic edema in the occipital and frontal lobe, suggestive of PRES. Her condition gradually improved after the treatment of hypercalcemia.

## Introduction

Posterior reversible encephalopathy syndrome (PRES) is a rare and severe neurotoxic encephalopathic state characterized by variable neurologic manifestations ranging from headache and confusion to seizures and coma. PRES was initially described in 1996, and the prevalence of the disease is reported to be as low as 0.03% [1]. Owing to the incomplete understanding of the mechanism of the disease and its nonspecific presentation, the terminology has been changing over the past two decades, as it has also been referred to as posterior leukoencephalopathy syndrome, hyperperfusion encephalopathy, and brain capillary leak syndrome [2]. As the original widely accepted name implies, this condition is mainly reversible and affects the occipital lobe of the brain, leading to a variety of nonspecific acute neurologic symptoms in both adult and pediatric populations such as seizures, headaches, and visual changes. Several diseases have been reported to increase the risk of developing PRES, such as hypertension, chronic kidney disease, and preeclampsia, and chemotherapeutic drugs have also been reported to be associated with PRES [3,4]. PRES induced by hypercalcemia is uncommon and not widely underlined in the literature [5-10]. We highlight a case of malignant hypercalcemia-induced PRES in a female patient diagnosed with advanced breast carcinoma.

## Case presentation

A 61-year-old female was brought to the emergency department in an unconscious state. She was diagnosed with advanced breast carcinoma of the right breast two months ago and was on hormonal treatment (tamoxifen) and targeted therapy (trastuzumab). She had multiple hospital visits for appetite loss, weight loss, severe backache, and gastrointestinal complaints. She had no previous history of hypertension or diabetes and reported no history of smoking, alcohol abuse, or illicit drug use. She had not taken any other medications except for backache and cancer therapy.

On examination, she was febrile (100^o^F) and tachycardic. She was not oriented to time, place, or person with a blood pressure of 160/90 mmHg, respiratory rate of 19/minute, heart rate of 125/minute, and oxygen saturation of 94% on room air. Her Glasgow Coma Scale (GCS) was seven, with bilateral flaccid limbs and normal reflexes. Her pupils were sluggish to respond, and she had no signs of meningeal irritation or a history of seizure episodes. Initial laboratory evaluations revealed severe hypercalcemia and liver and renal dysfunctions (Table 1).

**Table 1:** Results of initial blood investigations

Parameter	Lab value	Reference range
White cell count	9100/mm^3^	(4000-11000)
Hemoglobin	12 g/dl	(13-16)
Platelet count	161,000/mm^3^	(150,000-450,000)
Serum creatinine	3.1 mg/dl	(0.7-1.3)
Total serum calcium	16.9 mg/dl	(9.0-10.6)
Serum albumin	4.1 g/dl	(3.5-4.5)
Corrected calcium level	16.8	(9.0-10.5)
Blood urea nitrogen	101 mg/dl	(08-24)
Alanine aminotransferase	61 IU/L	(8-57)
Alkaline phosphatase	339 mg/dl	(36-95)
C-reactive protein	1.4 mg/dl	(0.3-1)
Erythrocyte sedimentation rate	22/hour	(0-20)
Lactate dehydrogenase	401 IU/L	(140-280)
Gamma-glutamyl transpeptidase	144 IU/L	(5-40)
Serum magnesium	1.7 mg/dl	(1.7-2.2)
Serum phosphorus	4.1 mg/dl	(2.8-4.5)
Parathyroid hormone	18 pg/ml	(10-55)

**Figure 1:**
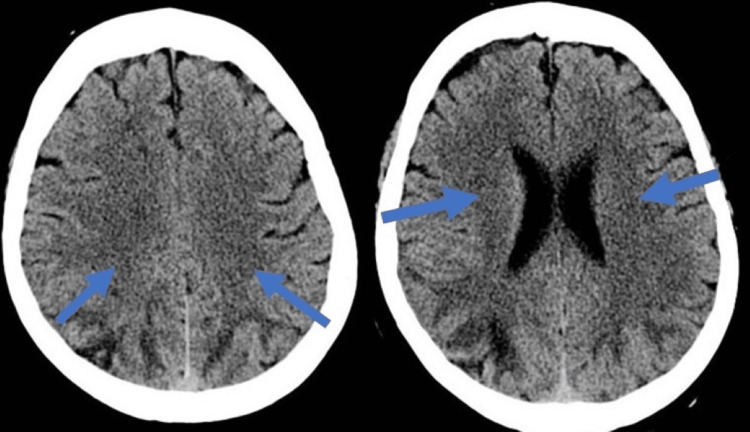
CT brain showing diffuse edema in the periventricular region of both cerebral hemispheres

Other investigations include thyroid function tests, vitamin D, and calcitonin, which were within normal range except for intact parathyroid hormone, which was in the lower normal range. An urgent electrocardiogram (ECG) showed sinus tachycardia with no QT abnormality. The chest X-ray was normal. Urgent brain computed tomography (CT) was performed, which showed diffuse edema in the bilateral parietal, occipital, and frontal lobes (Figure 1).

**Figure 2:**
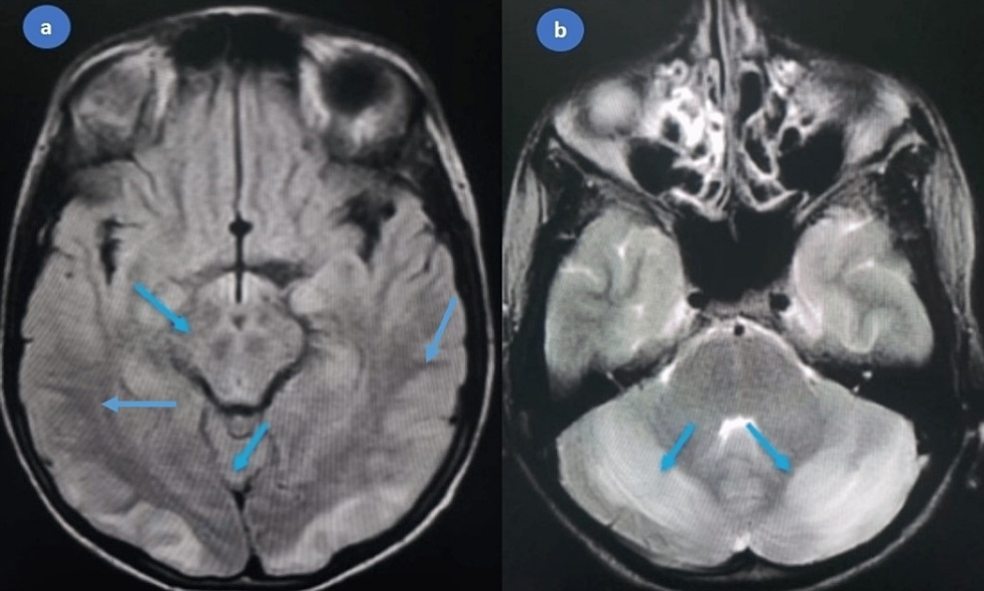
(a) MRI demonstrating hyperintensity on FLAIR, (b) T2 images involving bilateral parieto-occipital and medullary regions, mainly involving the cortical and subcortical areas and cerebral peduncles FLAIR: fluid-attenuated inversion recovery

**Figure 3:**
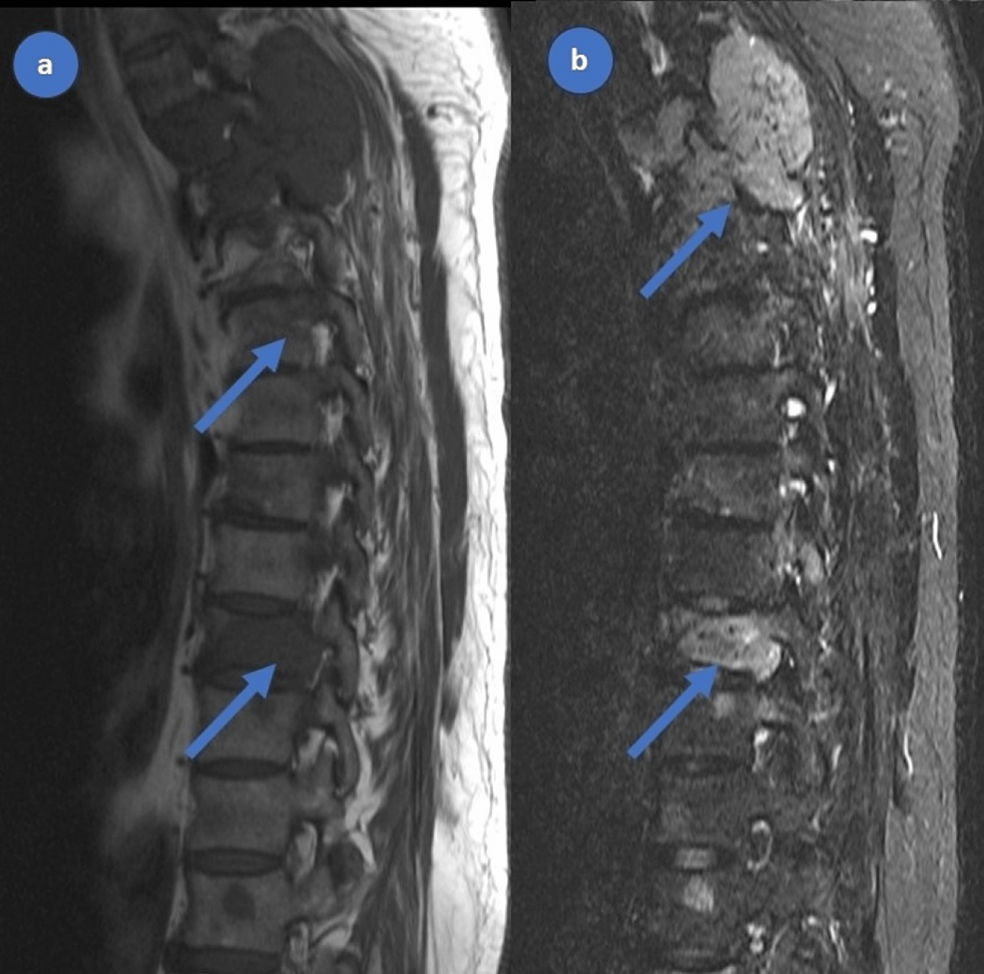
MRI spine demonstrating multiple lesions of the spine, involving vertebral bodies, posterior elements, and paraspinal soft tissue components (a, b)

She underwent a cerebrospinal fluid (CSF) examination, demonstrating normal cell cytology. Magnetic resonance imaging (MRI) of the brain revealed hyperintense lesions on T2 and fluid-attenuated inversion recovery (FLAIR) images involving bilateral high frontal and parieto-occipital regions, predominantly involving the cortical and subcortical areas, bilateral cerebral hemispheres, and medulla (Figure 2). Whole body MRI revealed extensive metastatic lesions mainly involving the vertebra (Figure 3).

She was managed with aggressive hydration, intravenous nicardipine, furosemide, and zoledronic acid. Cardiac telemetry was attached to monitor cardiac arrhythmias. After two days of treatment, her blood pressure was 130/85 mmHg, with a modest fall in serum calcium to 12.8 mg/dl. She started improving her consciousness level, and improvement in kidney and liver functions was observed. Her serum calcium level was 9.8 after nine days of treatment, and a repeat brain MRI was unremarkable. Based on her clinical, laboratory, and radiological findings, a provisional diagnosis of PRES induced by hypercalcemia was made.

## Discussion

PRES is a rare condition that is not widely reported except in case series and reports, and it can occur as a complication of specific diseases or drugs. PRES is commonly triggered by hypertension, eclampsia, chemotherapeutic drugs, and chronic kidney disease [8]. Hypercalcemia-induced PRES is rare, and a few cases of PRES triggered by hypercalcemia have been reported. We have tabulated the reported cases of hypercalcemia-induced PRES in Table 2 [5-10].

**Table 2:** Reported cases of hypercalcemia-induced PRES M: male, F: female PRES: posterior reversible encephalopathy syndrome

Author et al.	Age/Sex	Clinical Presentation	Etiology	Calcium (mg/dl)	Management
Bolanthakodi et al. [5]	62/F	Altered sensorium, seizures	Calcium overdose	18.3	Hydration, calcitriol
Camara-Lemarroy et al. [6]	38/F	Altered sensorium	Metastatic breast cancer	14.5	Hydration, zoledronate
Nakajima et al. [7]	58/M	Confusion, headache, visual disturbance	Metastatic esophageal cancer	17.5	Aggressive hydration, calcitonin
Moussawi et al. [8]	68/F	Visual disturbance, altered sensorium	Lymphoma	18	Hydration, calcitonin
Song et al. [9]	51/F	Low back pain	Metastatic breast carcinoma	15.8	Hydration, furosemide, bisphosphonate
Mirian et al. [10]	74/F	Altered sensorium, headache	Hyperparathyroidism	14	parathyroidectomy

The PRES pathophysiology is not entirely understood, and some theories have been put forward to explain it. The vasogenic theory (breakthrough or hyperperfusion theory) explains more than half of the cases. This theory suggests that rapidly increasing systematic blood pressure leads to endothelial damage and disruption of the cerebral vascular autoregulatory mechanisms, leading to hyperperfusion and dysfunction of the blood-brain barrier, which increases its permeability and ultimately causes vasogenic edematous changes [6]. However, the occipital lobe is mainly involved due to a lesser sympathetic innervation of other regions in the brain, like the frontal lobe [8,9]. It is, therefore, not surprising that PRES is commonly associated with hypertension and other diseases that lead to elevated blood pressure, such as renal failure and hypercalcemia, as in our case. The rest of the PRES cases are attributed to the direct cytotoxic effects of certain medications on the endothelium, like cyclosporine, tacrolimus, other chemotherapeutic agents, and autoimmune diseases [5]. PRES is a diagnosis of exclusion and neuroimaging using brain CT or MRI, and clinical history of neurologic symptoms and risk factors are essential components to support the diagnosis, as it can occasionally mimic stroke of the posterior circulation. Due to these nonspecific signs and symptoms, the differential diagnosis is broad and can include osmotic demyelination syndrome, acute demyelinating encephalomyelitis, infectious encephalitis, toxic leukoencephalopathy, cerebral sinus venous thrombosis, subdural or subarachnoid hemorrhage, and uremic encephalopathy [9,11].

The management of PRES is mainly to address the underlying cause. Rapidly recognizing and minimizing the risk factors is essential. The mainstay treatment of malignant hypercalcemia is volume resuscitation with normal saline to restore normal kidney function and calciuresis and maintain adequate urine output [4]. Diuretics can be used to promote calciuresis. Bisphosphonates are currently recommended against hypercalcemia due to the inhibition of osteoclastic bone resorption. Calcitonin can also be used to inhibit bone resorption and kidney calcium reabsorption [7-9]. Steroids can also be used in conjugation with standard therapy in patients with malignant hypercalcemia, as they inhibit osteoclastic bone resorption and reduce extrarenal vitamin D activity. In patients with severe hypercalcemia or where rapid correction of calcium is mandatory, hemodialysis using a calcium-free dialysate could be effective [12,13]. Our patient was diagnosed with PRES induced by hypercalcemia and responded well to hydration, diuresis, and bisphosphonates therapy.

## Conclusions

Although rare, hypercalcemia can induce PRES. PRES is a diagnosis of exclusion and should be considered among differential diagnoses in patients with severe hypercalcemia who presented with neurological manifestations such as headaches, altered sensorium, and seizures. A prompt diagnosis is essential for the excellent prognosis and timely correction of hypercalcemia, and managing the underlying condition is obligatory to prevent severe morbidity and mortality.
